# Luteolin increases susceptibility to macrolides by inhibiting MsrA efflux pump in *Trueperella pyogenes*

**DOI:** 10.1186/s13567-021-01021-w

**Published:** 2022-01-10

**Authors:** Yuru Guo, Chengcheng Huang, Hongyu Su, Zehui Zhang, Menghan Chen, Ruxia Wang, Dexian Zhang, Luyao Zhang, Mingchun Liu

**Affiliations:** Key Laboratory of Livestock Infectious Diseases in Northeast China, Ministry of Education, College of Animal Science and Veterinary Medicine, Shenyang Agricultural University, Shenyang, Liaoning China

**Keywords:** *Trueperella pyogenes*, luteolin, efflux pump inhibitors, MsrA efflux pump

## Abstract

*Trueperella pyogenes* (*T. pyogenes*) is an opportunistic pathogen associated with a variety of diseases in many domestic animals. Therapeutic treatment options for *T. pyogenes* infections are becoming limited due to antimicrobial resistance, in which efflux pumps play an important role. This study aims to evaluate the inhibitory activity of luteolin, a natural flavonoid, on the MsrA efflux pump and investigate its mechanism. The results of antimicrobial susceptibility testing indicated that the susceptibility of *msrA*-positive *T. pyogenes* isolates to six macrolides increased after luteolin treatment, while the susceptibility of *msrA*-negative isolates showed no change after luteolin treatment. It is suspected that luteolin may increase the susceptibility of *T. pyogenes* isolates by inhibiting MsrA activity. After 1/2 MIC luteolin treatment for 36 h, the transcription level of the *msrA* gene and the expression level of the MsrA protein decreased by 55.0–97.7% and 36.5–71.5%, respectively. The results of an affinity test showed that the equilibrium dissociation constant (KD) of luteolin and MsrA was 6.462 × 10^–5^ M, and hydrogen bonding was predominant in the interaction of luteolin and MsrA. Luteolin may inhibit the ATPase activity of the MsrA protein, resulting in its lack of an energy source. The current study illustrates the effect of luteolin on MsrA in *T. pyogenes* isolates and provides insight into the development of luteolin as an innovative agent in combating infections caused by antimicrobial-resistant bacteria.

## Introduction

*Trueperella pyogenes* (*T. pyogenes*), formerly classified as *Corynebacterium pyogenes*, is a gram-positive, non-spore-forming, nonencapsulated, nonmotile, pleomorphic, rod-shaped bacterium [1–3]. *T. pyogenes* is an opportunistic and zoonotic pathogen. In domestic and wildlife animals, it often causes various suppurative infections, including liver abscess, arthritis, pneumonia, metritis, and mastitis [4, 5]. Infections caused by *T. pyogenes* in humans are rare and usually occur with occupational exposure, especially in individuals in contact with animals and their environment. It has been reported that *T. pyogenes* causes endocarditis, pneumonia, arthritis, and various purulent lesions and abscesses in humans [3, 6].

*Trueperella pyogenes* is an important causative agent in cattle and pigs due to the fact that it causes substantial economic losses [1]. At present, the treatment of infections caused by *T. pyogenes* in veterinary clinics still relies on antibiotics [7–9]. However, the antimicrobial resistance of *T. pyogenes* is becoming severe because of the overuse of antimicrobial drugs in veterinary clinical treatment. Zhang reported that *T. pyogenes* isolated from dairy cows was highly resistant to tetracycline and doxycycline [10]. High MIC_90_ values for oxytetracycline (32 μg/mL), tylosin (64 μg/mL) and erythromycin (1024 μg/mL) have been observed among *T. pyogenes* isolates from ruminants [11]. Most *T. pyogenes* isolates from domestic animals and European bison were nonsusceptible to enrofloxacin and ciprofloxacin [7]. Antimicrobial resistance, especially multidrug resistance, seriously threatens the therapeutic efficiency of antimicrobial agents. To cope with excessive resistance in bacteria, it is urgent to develop new drugs to combat this resistance.

Bacteria can develop resistance to antibiotics through various mechanisms: (i) reducing the permeability of the cell membrane, (ii) pumping antibiotics out of the cells via efflux pump systems, (iii) expressing enzymes that can destroy or modify antibiotics, and (iv) modifying the specific target of antibiotics [12, 13]. There are five major families of bacterial efflux pumps, among which ATP-binding cassette (ABC) family transporters can be found in both gram-positive and gram-negative bacteria. The macrolide resistance gene *msrA*, which encodes an efflux pump, is prevalent in *Staphylococcus aureus*, *Staphylococcus epidermidis* and other staphylococcal species. As a member of the ABC transporters, the MsrA pump confers resistance to 14- and 15-membered ring macrolides and streptogramin B [14].

Identifying effective efflux pump inhibitors (EPIs) that prevent bacteria from pumping antibiotics out of the cell contributes to improving the clinical performance of antibiotics [15]. Many EPIs have been identified, such as β-naphthylamide, carbonyl cyanide-*m*-chlorophenylhydrazone, verapamil, and reserpine [16]. However, most of these molecules are limited in clinical application due to their toxicity, instability and low bioavailability [17–20]. Recently, interest has been growing in the identification of EPIs from natural products, including flavonoids. It has been reported that baicalein could improve the susceptibility of *Staphylococcus aureus* to antibiotics by inhibiting NorA and TetK efflux pumps [21]. The flavonoids skullcapflavone II and nobiletin have been found to interfere with efflux pump activity in *M. aurum* and *M. smegmatis* [22].

However, studies carried out to evaluate the effect of natural products on the MsrA efflux pump are scarce. Luteolin, a typical flavonoid, has many pharmacological activities, such as antibacterial, anti-inflammatory, antioxidant, and antitumour activities [23, 24]. In addition, our previous study showed that luteolin could increase the susceptibility of multidrug-resistant *T. pyogenes* to aminoglycoside antibiotics by inhibiting the MATE efflux pump [25]. This study aims to explore the potential activity of luteolin as an MsrA inhibitor and its mechanism of action.

## Materials and methods

### Strains and culture conditions

*Trueperella pyogenes* isolates were obtained from cows suffering from endometritis and preserved in our laboratory. Isolates were classified as *msrA*-positive or *msrA*-negative strains by PCR amplification of the *msrA* gene in our previous study. Eight *msrA*-positive *T. pyogenes* isolates (HC03-1, HC-H03-3, HC-H02-2, BM-07-1, BM-H06-3, BM-H11-1, BM-H01-1 and RY04-2) and two *msrA*-negative *T. pyogenes* isolates (HC-H10 and RY14-3) were included in this study. *T. pyogenes* isolates were grown on Mueller–Hinton agar (MH(A), AOBOX, Beijing, China) supplemented with 5% sterile defibrinated sheep blood (Solarbio, Beijing, China) for 48 h at 37 °C in 5% CO_2_. Before assays, 3–5 colonies were inoculated into nutrient broth (NB, AOBOX, Beijing, China) containing 8% foetal bovine serum (FBS, Gibco, USA) and cultured in tubes for 24 h at 37 °C with shaking at 180 rpm to obtain a bacterial suspension that reached the logarithmic phase.

### Antimicrobial agents and chemicals

Macrolides, including erythromycin, roxithromycin, acetylspiramycin, tilmicosin, azithromycin and tylosin, were obtained from the China Institute of Veterinary Drug Control. Luteolin was purchased from Shanghai Pureone Biotechnology Co., Ltd. (Shanghai, China) and dissolved in 1% dimethyl sulfoxide (DMSO, Sigma–Aldrich, Shanghai, China) to produce a stock solution. Reserpine was purchased from Chengdu Must Bio-technology Co., Ltd. (Chengdu, China) and dissolved in 1% DMSO to produce a stock solution. Reserpine was used as a test control due to its previously demonstrated action as an EPI [26].

### Antimicrobial susceptibility testing

Minimum inhibitory concentrations (MICs) of luteolin and reserpine against *T. pyogenes* isolates were determined by the broth microdilution method according to Clinical and Laboratory Standards Institute (CLSI) guidelines, with the following modification [27]. Briefly, 100 μL of serial twofold dilutions of luteolin and reserpine in Mueller–Hinton broth (MH(B), AOBOX, Beijing, China) containing 8% FBS were dispensed in U-bottom 96-well microtitre plates (Corning, USA). An equal volume of adjusted inoculum (5 × 10^5^ CFU/mL) was added to each well of the microplate up to a final volume of 200 μL. The microtitre plates were observed after 24 h incubation at 37 °C with 5% CO_2_. The MICs were defined as the lowest concentrations of luteolin and reserpine that prevented visible bacterial growth.

The effects of luteolin on the susceptibility of *T. pyogenes* to six macrolides were subsequently investigated. Briefly, a *T. pyogenes* suspension cultured to the logarithmic phase was adjusted to a final concentration of 5 × 10^5^ CFU/mL and mixed with different final concentrations of luteolin (1/2 MIC, 1/4 MIC, 1/8 MIC, and 1/16 MIC). The same final concentration of DMSO (v/v, 0.01%) was added as a solvent control. The mixture was incubated in tubes at 37 °C and 180 rpm for 18, 24, 30, 36, 42, 48, 54, and 60 h. Then, 30 μL of mixed cultures of *T. pyogenes* and luteolin were inoculated into 3 mL of MH(B) containing 8% FBS and cultured to the logarithmic phase. The changes in the MICs of macrolides against *T. pyogenes* after luteolin treatment were determined following the broth microdilution method. The same method was used to analyse the effect of reserpine on the susceptibility of *T. pyogenes* to macrolides. *Staphylococcus aureus* ATCC29213 was included as a quality control strain in all antimicrobial susceptibility assays, and MIC data were accepted only if the MIC of the control strain was within the required reference ranges. The determinations were performed in triplicate.

### Analysis of the expression of *msrA* using quantitative real-time PCR

The effects of luteolin on the transcription level of the *msrA* gene were determined by quantitative real-time PCR (qRT–PCR). *T. pyogenes* cultured to the logarithmic phase was diluted to 1 × 10^5^ CFU/mL and mixed with luteolin (final concentration, 1/2 MIC). The same method was used to treat *T. pyogenes* with reserpine at a final concentration of 1/2 MIC. The same final concentration of DMSO (v/v, 0.01%) was added as a solvent control. After the bacterial suspension was incubated at 37 °C and 180 rpm for 36 h, 50 mL of culture was harvested to collect bacterial cells by centrifugation for 2 min at 12 000 rpm and 4 °C. Total RNA of *T. pyogenes* was extracted using TRIzol Reagent (Ambion, Carlsbad, USA), and cDNA was synthesized using a PrimeScript™ RT Reagent Kit with gDNA Eraser (TaKaRa, Dalian, China).

The transcription level of the *msrA* gene was measured using a TB Green® *Premix Ex Taq*™ II Kit (TaKaRa, Dalian, China) on an Applied Biosystems® QuantStudio® 3 Real-Time PCR System (Thermo Fisher, USA) according to the manufacturer’s instructions. The *16S rRNA* of *T. pyogenes* was used as a reference gene for comparison in qRT–PCR. The primer sequences are shown in Table 1. The qRT–PCR conditions were set as follows: initial denaturation at 95 °C for 3 min; 40 cycles of 95 °C for 10 s, 60 °C for 10 s, and 72 °C for 30 s; followed by a melting curve at 95 °C for 15 s, 60 °C for 60 s, and finally 95 °C for 1 s. The relative expression of the *msrA* gene was normalized against that of *16S rRNA* and quantified using the 2^−ΔΔCt^ method. Three independent experiments were carried out in duplicate.

**Table 1:** **Primer sequences used in qRT–PCR assays**

Gene	Orientation	Sequence (5′-3′)
*msrA*	Forward	GGCATACTATCGTCAACTTG
	Reverse	ATACTGCTAACGATAATTTCG
*16S rRNA*	Forward	ATGCAACGCGAAGAACCTTACC
	Reverse	TTAACCCAACATCTCACGACAC

### Preparation of a polyclonal antibody against His-MsrA protein

The coding sequence of *msrA* was amplified from the genomic DNA of strain BM-H06-3. Primers were designed based on the multiple cloning site of the pET-28a vector. The primers and restriction sites are shown in Table 2. The PCR product of *msrA* was ligated with the linearized pET-28a vector to generate the recombinant plasmid pET-28a-*msrA*, which was then transformed into *E. coli* BL21 (DE3) cells. The bacteria were cultured in LB medium supplemented with 50 μg/mL kanamycin. When the cell density of BL21 (DE3) reached 0.4–0.6 at OD_600 nm_, 1 mM isopropyl-β-d-thiogalactoside (IPTG, Sigma, USA) was added to the medium to induce the expression of His-MsrA fusion protein at 16 °C for 12 h [28]. The bacterial cells induced by IPTG were harvested, resuspended in PBS (pH 7.4) and lysed by sonication on ice (3 s on and 3 s off, total of 20 min). After sonication, the total proteins existed in the supernatant after centrifugation at 4 °C and 12 000 rpm for 10 min. The His-MsrA protein was purified using a His GraviTrap (GE Healthcare, Sweden) according to the manufacturer’s recommendations. The purified protein was identified by Western blotting analysis using an anti-His mouse monoclonal antibody (TransGen Biotech, Beijing, China) and HRP-conjugated goat anti-mouse IgG (TransGen Biotech, Beijing, China) [29].

**Table 2:** **Primer sequences used in the expression of the His-MsrA fusion protein**

Gene	Orientation	Sequence (5′-3′)	Restriction site
*msrA*	Forward	GGATCCATGGAACAATATACAATTAAATTTAACCAAATCA	BamH I
	Reverse	AAGCTTTTAAGTTATATCATGAATAGATTGTCCTGTTAATTCC	Hind III

The purified His-MsrA fusion protein was used to produce a polyclonal antibody. Briefly, adult New Zealand white rabbits were immunized by the subcutaneous route with 400 μg of His-MsrA protein mixed with an equal volume of complete Freund’s adjuvant (Sigma–Aldrich, Shanghai, China) at the first injection. Two weeks later, the rabbits were given three booster injections of 400 μg of fusion protein with incomplete Freund’s adjuvant (Sigma–Aldrich, Shanghai, China) every week. One week later, the rabbits were bled to collect the antiserum [30]. The purified fusion protein was used as a coating antigen to detect the titres of the anti-His-MsrA polyclonal antibody by indirect enzyme-linked immunosorbent analysis (ELISA).

### Extraction of *T. pyogenes* total proteins and Western blotting analysis of MsrA

The treatment method of *T. pyogenes* samples was the aforementioned method in “Analysis of the expression of *msrA* using quantitative real-time PCR”. After the bacterial suspension was incubated on a shaker at 37 °C and 180 rpm for 36 h, 100 mL of the bacterial suspension was centrifuged at 12 000 rpm and 4 °C for 2 min. The bacterial cells were fully mixed with 2 mL of bacterial protein extraction reagent (CWBIO, Beijing, China) and 20 μL of protease inhibitor cocktail (CWBIO, Beijing, China). The mixture was sonicated on ice (3 s on and 3 s off, total of 20 min) and centrifuged at 12 000 rpm for 10 min at 4 °C to acquire the total cell proteins. The protein concentration was measured using a BCA protein assay kit (Thermo Fisher, USA).

The protein expression level of MsrA after luteolin treatment was determined by Western blotting. Briefly, the total proteins from *T. pyogenes* were run on a 12% SDS–PAGE gel and then transferred onto PVDF membranes (Millipore, Germany). After blocking with 5% skim milk in TBST buffer at 4 °C for 12 h, membranes were incubated with the anti-His-MsrA polyclonal antibody at a dilution of 1:5000 for 2 h at 37 °C. Then, membranes were washed three times with TBST buffer for 10 min each time and incubated with HRP-conjugated goat anti-rabbit IgG (1:10 000) (Sangon, Shanghai, China), which was used as the secondary antibody, at 37 °C for 2 h. An anti-GAPDH mouse monoclonal antibody (Sangon, Shanghai, China) was used to detect the expression of the reference protein, and HRP-conjugated goat anti-mouse IgG (Sangon, Shanghai, China) was used as the secondary antibody [31]. Finally, after being washed three times with TBST buffer as above, the membranes were visualized using an *EasySee*® Western Blot Kit (TransGen Biotech, Beijing, China) and an Azure c300 imaging system (Azure, USA).

### Affinity test of luteolin to MsrA

To explore the interaction between luteolin and the MsrA protein, the affinity of luteolin to the MsrA protein was tested using surface plasmon resonance (SPR) with a Biacore T200 system (GE Healthcare, Sweden). Briefly, the fusion protein His-MsrA was captured on an NTA sensor chip (GE Healthcare, Sweden) by chelation of Ni^2+^ through NTA on the surface and histidine residues in the ligand tag. Luteolin was diluted with PBS-P (GE Healthcare, Sweden) to a series of concentrations (12.5, 25, 50, 100, 200, and 400 μM). The luteolin solution was flowed at a rate of 30 μL/min for 180 s to allow for association, followed by 300 s for dissociation over captured protein in PBS-P running buffer [32]. The binding ability of luteolin to the His-MsrA protein was evaluated by the equilibrium dissociation constant (KD) using Biacore T200 Evaluation Software 3.0.

### Structure prediction of MsrA and docking analysis

Homology modelling was used to predict the tertiary structure of the MsrA protein. The protein sequence was submitted to the online protein structure homology-modelling website [33], and then homologous proteins with high sequence homology were searched in the database for each amino acid sequence. Using these proteins with known crystal structures as templates, a three-dimensional (3D) structure model of the target protein was constructed. The protein with the highest homology was selected to construct the 3D structure of the target protein, which was saved in PDB file format for subsequent molecular docking. For ligand preparation, the structure of luteolin was drawn using ChemDraw Ultra 7.0, energy-minimized in Chem3D Ultra 7.0 and saved as a PDB file. AutoDock is a suite of tools used to predict interactions between small molecules (ligands) and proteins of known 3D structure based on the properties (polar atoms and bond rotations) of the ligand and the binding site of the protein [34]. AutoDock 4.2 was used for the molecular docking of luteolin and MsrA protein.

### Statistical analysis

The results of qRT–PCR and Western blotting are expressed as the mean ± standard deviation (SD) of three independent experiments. Statistical analysis was performed by GraphPad Prism (scientific 2D graphing and statistics software, version 6.0, California Corporation) and one-way analysis of variance (ANOVA) in SPSS Statistics (IBM Company, version 17.0, SPSS Inc., New York, USA). Statistical significance was defined as */#*P* < 0.05 and **/##*P* < 0.01.

## Results

### Luteolin increases the susceptibility of *T. pyogenes* to macrolides

The minimum inhibitory concentrations (MICs) of luteolin and reserpine against *T. pyogenes* are presented in Table 3. The MICs of luteolin against each isolate were all 78 μg/mL except BM-H06-3 (156 μg/mL), regardless of whether these strains carried efflux pumps. The MICs of reserpine, a test control, against *T. pyogenes* strains ranged from 5 to 40 μg/mL.

**Table 3:** **The MIC of luteolin against** ***T. pyogenes*** ***(μg/mL)***

Drug name	Strain number									
	HC03-1	HC-H03-3	HC-H02-2	BM07-1	BM-H06-3	BM-H11-1	BM-H01-1	RY04-2	HC-H10	RY14-3
Luteolin	78	78	78	78	156	78	78	78	78	78
Reserpine	5	5	5	5	20	20	20	20	20	40

**Figure 1:**
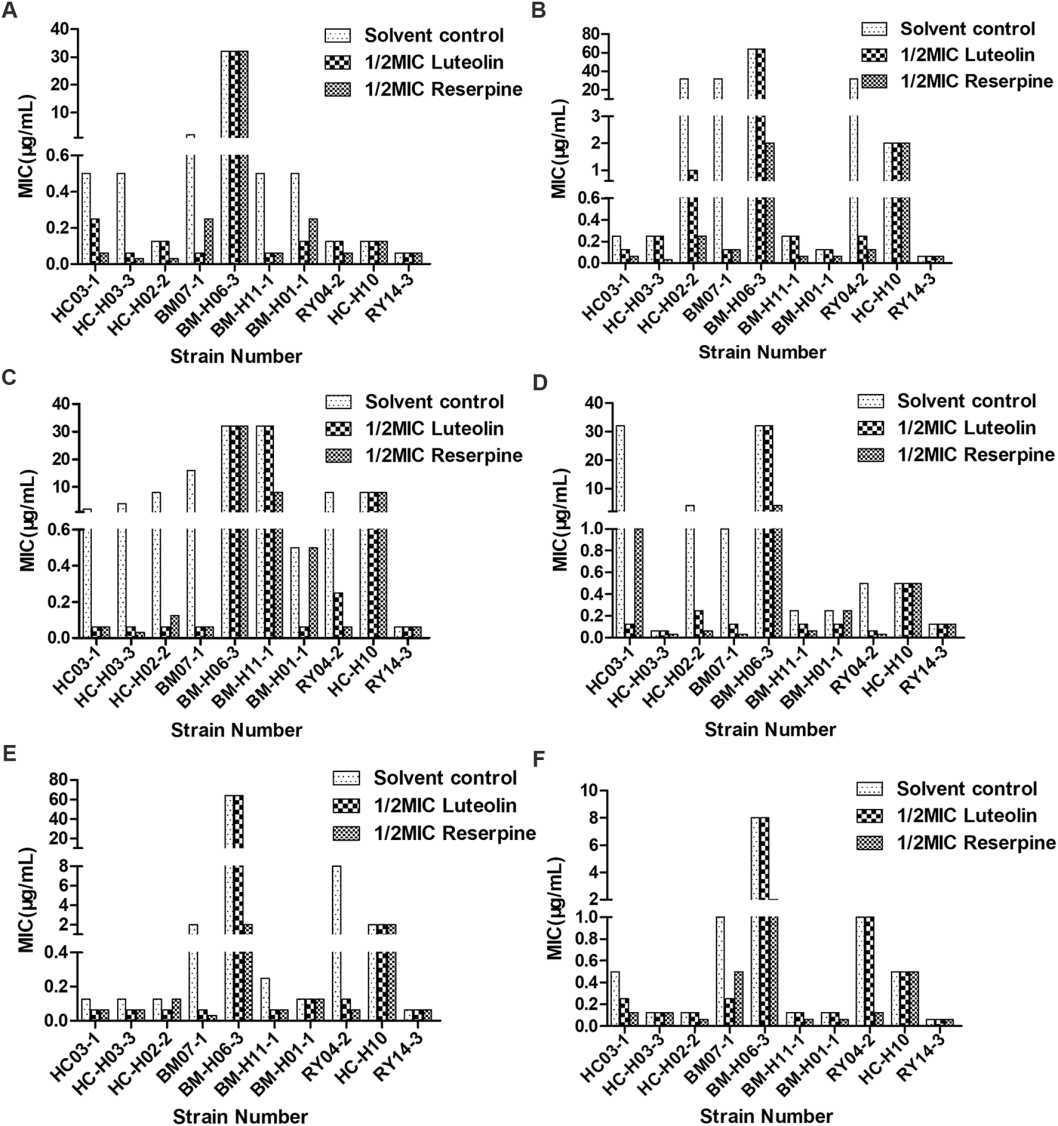
**The changes in the MICs of six macrolides against *T. pyogenes* after luteolin treatment.** **A** Erythromycin, **B** roxithromycin, **C** acetylspiramycin, **D** tylosin, **E** azithromycin, **F** tilmicosin.

It was found that 1/2 MIC luteolin treatment for 36 h could best increase the susceptibility of *T. pyogenes* to macrolides. After treatment with luteolin at 1/2 MIC, the MICs of macrolides against *T. pyogenes* decreased by 1-256-fold, and a similar situation occurred in isolates treated with reserpine (shown in Figure 1). Among eight *msrA*-positive *T. pyogenes* isolates (HC03-1, HC-H03-3, HC-H02-2, BM-07-1, BM-H06-3, BM-H11-1, BM-H01-1 and RY04-2), six isolates showed increased susceptibility to acetylspiramycin, tylosin and azithromycin (Figures 1C–E), five isolates showed increased susceptibility to erythromycin (Figure 1A), four isolates showed increased susceptibility to roxithromycin (Figure 1B), and only two isolates showed increased susceptibility to tilmicosin (Figure 1F). Conversely, after luteolin treatment, the MICs of six macrolides against *msrA*-negative *T. pyogenes* isolates (HC-H10 and RY14-3) showed no changes, which indicated that luteolin may influence the MsrA efflux pump activity in *T. pyogenes*, leading to a decrease in macrolide resistance.

### Effects of luteolin on the transcription level of the *msrA* gene

**Figure 2:**
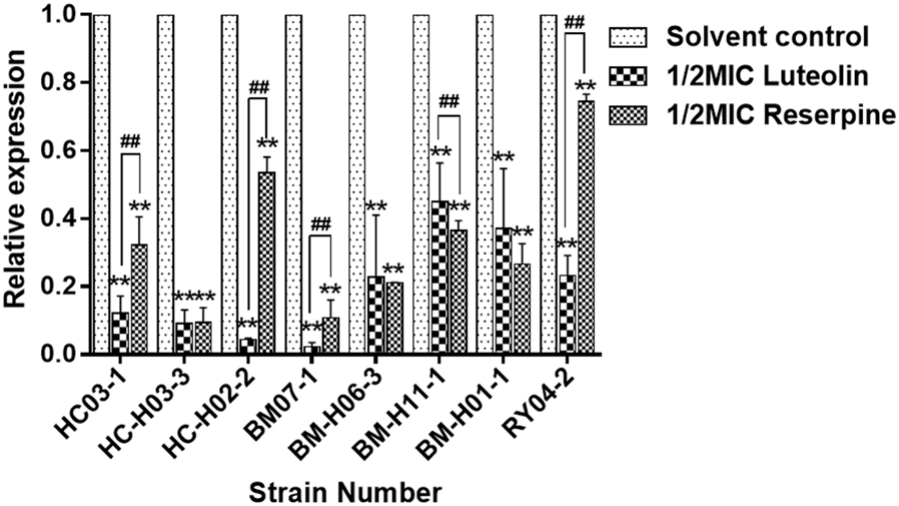
**The relative expression level of the *msrA* gene after luteolin treatment.** Data were presented as means ± SDs (*compared with solvent control, #compared with reserpine, */# *P* < 0.05, **/## *P* < 0.01).

qRT–PCR was used to investigate the effects of luteolin on the transcription level of the *msrA* gene. After luteolin and reserpine treatment, the relative mRNA expression of the *msrA* gene showed different degrees of reduction in all *T. pyogenes* strains that produced the MsrA pump (Figure 2). Compared with the solvent control group, the expression of the *msrA* gene decreased by 55.0–97.7% in the luteolin treatment group and 25.5–90.6% in the reserpine treatment group. The expression of the *msrA* gene in BM07-1 was the lowest after luteolin treatment. In the reserpine treatment group, the relative expression of the *msrA* gene in HC-H03-3 was the lowest.

### Effects of luteolin on the protein expression level of MsrA

**Figure 3:**
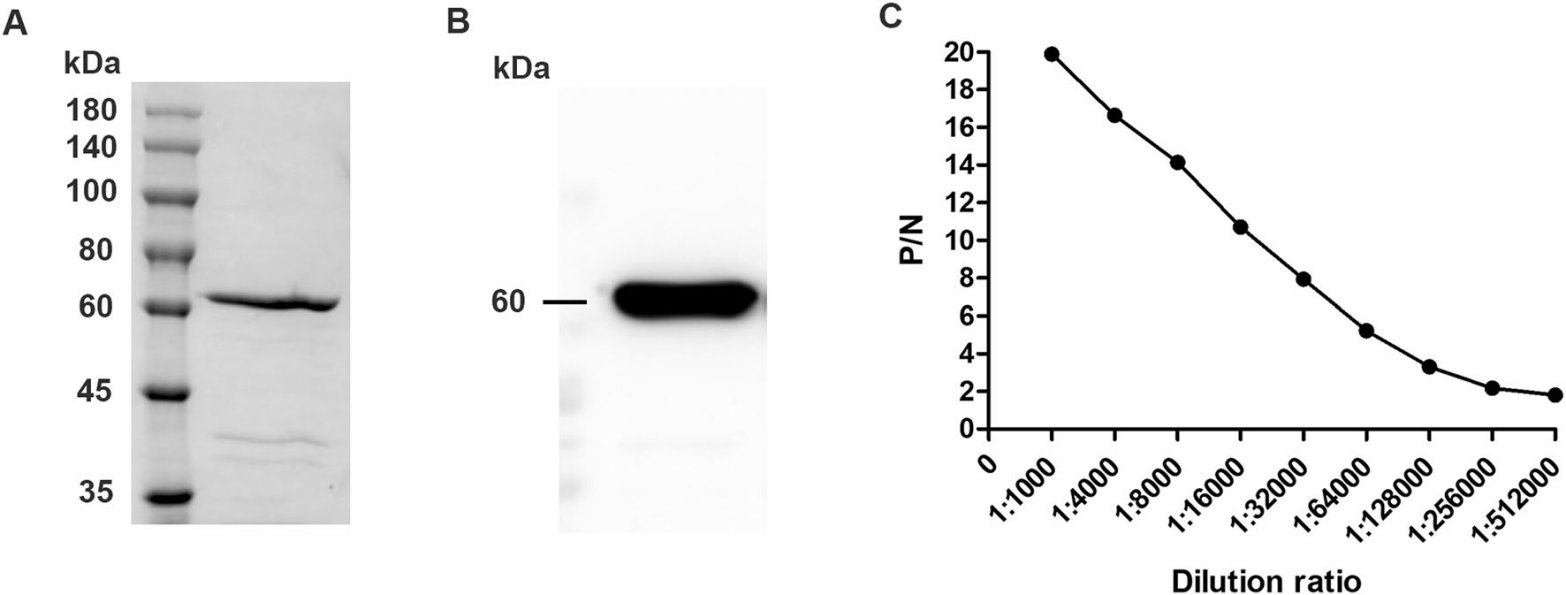
**Preparation of the anti-His-MsrA polyclonal antibody.** **A** SDS–PAGE analysis of purified His-MsrA fusion protein. **B** Western blotting analysis of purified His-MsrA fusion protein using an anti-His monoclonal antibody. **C** Antiserum titre determination by ELISA.

**Figure 4:**
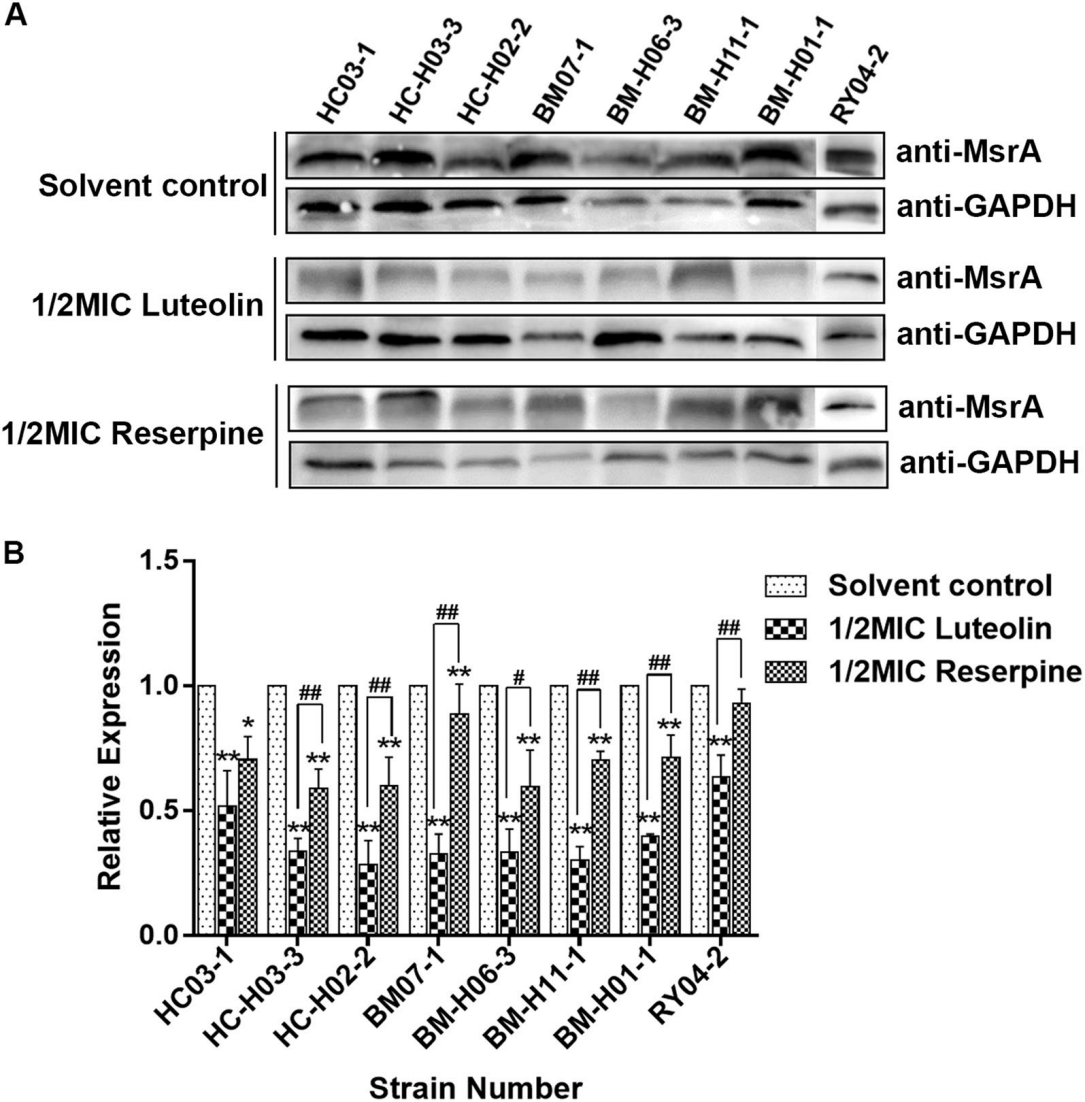
**The effect of luteolin on the expression level of the MsrA protein.** **A** Western blotting results of the MsrA protein in *T. pyogenes* after luteolin treatment. The experiment was repeated separately three times, and one representative blot is shown. **B** The relative expression level of the MsrA protein after luteolin treatment. Data were presented as means ± SDs (*compared with solvent control, #compared with reserpine, */#*P* < 0.05, **/##*P* < 0.01).

To investigate the effect of luteolin on the protein expression level of MsrA, polyclonal antibodies against His-MsrA were prepared by immunizing New Zealand white rabbits. The purification and identification results of the His-MsrA fusion protein are shown in Figures 3A and B, which indicated that the His-MsrA protein was successfully purified. The ELISA results showed that the titre of the anti-His-MsrA polyclonal antibody reached 1:256 000, which showed that the rabbit antiserum was expected to successfully recognize the MsrA protein of *T. pyogenes* (Figure 3C). After obtaining the anti-His-MsrA polyclonal antibody, the effects of luteolin on the protein expression of MsrA in *T. pyogenes* were analysed by Western blotting assays. Western blotting assays of MsrA protein were repeated three times, and one representative blot is shown (Figure 4A). As shown in Figures 4B, among all *msrA*-positive *T. pyogenes* isolates, the relative expression of MsrA decreased by 36.5–71.5% after 1/2 MIC luteolin treatment for 36 h. Similarly, reserpine also significantly reduced the protein expression level of MsrA (decreased by 6.9–41.2%). However, luteolin caused a lower expression level of MsrA than reserpine in all *msrA*-positive *T. pyogenes* isolates.

### The affinity of luteolin and MsrA protein

**Figure 5:**
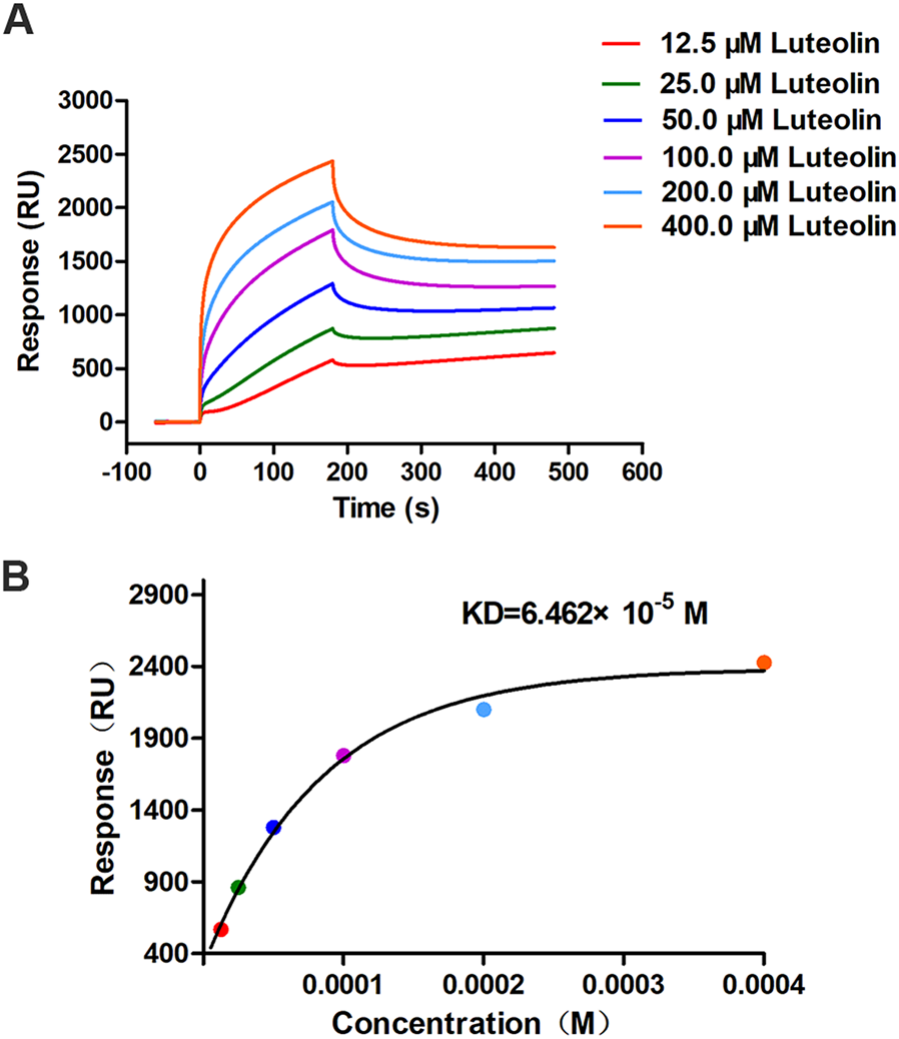
**Detection of the interaction between luteolin and the MsrA protein by SPR.** **A** Affinity sensing diagram for a series of concentrations of luteolin with MsrA protein. **B** Affinity fitting curve of luteolin with the MsrA protein.

To verify whether luteolin could interact with MsrA, the affinity was assessed using surface plasmon resonance (SPR) with a Biacore T200 system. The sensor diagram and fitting curve are shown in Figure 5. As shown in Figure 5A, luteolin and MsrA protein combined at 0–180 s and dissociated at 180–480 s. The KD value for the interaction between luteolin and MsrA protein was 6.462 × 10^–5^ M (Figure 5B), representing a reliable affinity between luteolin and the MsrA protein. The results of the affinity assay demonstrated that luteolin can bind with MsrA, which was further verified by molecular docking.

### Molecular modelling of the MsrA protein and docking analysis

**Figure 6:**
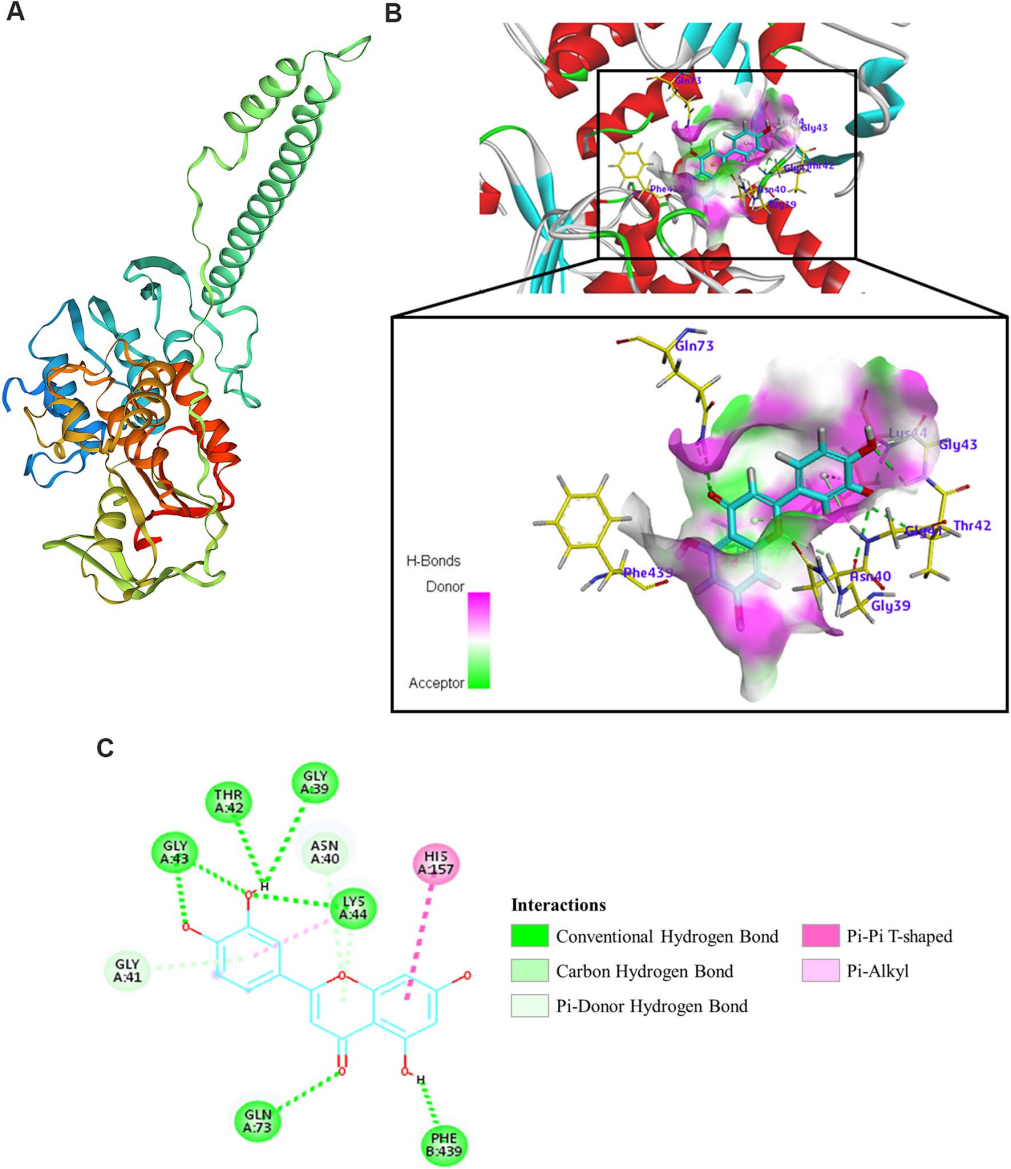
**The molecular docking results of luteolin and the MsrA protein.** **A** Predicted 3D structure model of the MsrA protein. **B** Luteolin interacting with the amino acid residues located within the active site of MsrA in a 3D docking model. **C** Molecular interactions between luteolin and MsrA in a 2D docking model.

The AutoDock program was used to predict the interaction site of luteolin with the MsrA protein. First, the 3D structure of the MsrA protein was generated after the amino acid sequence was submitted to the online protein structure prediction website. The predicted model was obtained based on the protein sequence of MsrA as well as on amino acid sequence alignment and structure comparison. The predicted model of MsrA (SMTL ID: 6ha8.1) is shown in Figure 6A, and the three-dimensional structure of MsrA was exported in PDB file format. The 3D docking results of luteolin with MsrA showed that luteolin lies in the hydrophobic pocket of the MsrA active site (Figure 6B) and interacts with Gly39, Asn40, Gly41, Thr42, Gly43, Lys44, Gln73, His157 and Phe439 (Figure 6C). Among these amino acid residues, most of them (Gly39, Asn40, Gly41, Thr42, Gly43 and Lys44) belong to ATP-binding and ATPase active regions. Therefore, it is predicted that luteolin may inhibit the efflux of drugs by preventing the MsrA efflux pump from obtaining energy. The two-dimensional (2D) docking results showed that luteolin interacted with MsrA mainly through conventional hydrogen bonds and carbon hydrogen bonds (Figure 6C).

## Discussion

Identifying compounds that inhibit efflux pumps is a promising method to combat resistant bacteria. Many studies have reported that flavonoids could reverse the resistance of bacteria. For example, silybin could restore the susceptibility of methicillin-resistant *Staphylococcus aureus* (MRSA) to ciprofloxacin by inhibiting the expression of efflux genes and proteins [35]. Belofsky reported that a new flavonoid could potentiate the activity of antimicrobials through inhibition of NorA multidrug resistance (MDR) pumps [36]. Additionally, hydnocarpins and their derivatives showed efficient inhibitory activity on the NorA efflux pump of *Staphylococcus aureus* and its increased susceptibility to enrofloxacin [37].

Previous studies have shown that luteolin exhibits multiple pharmacological effects, such as antibacterial, anti-inflammatory, antioxidant, and antitumour effects [23, 24], but the effect of luteolin on efflux pumps is still unclear. In this study, the MICs of six macrolides against *T. pyogenes* after luteolin treatment were analysed. We found that treatment with luteolin at a 1/2 MIC concentration for 36 h could maximize the susceptibility of *msrA*-positive *T. pyogenes* isolates to macrolides. However, the susceptibility of *msrA*-negative *T. pyogenes* isolates to macrolides showed no changes after luteolin treatment, which indicated that luteolin increased the susceptibility of *T. pyogenes* by acting on MsrA. Reserpine has been widely studied as a general inhibitor of efflux pumps. Although this molecule can inhibit the ABC transporters involved in the extrusion of antibiotics, the concentrations necessary to block bacterial efflux are neurotoxic [38]. In this study, luteolin, known to have many pharmacological activities and almost no toxicity [39], had the same effect as reserpine in reversing the resistance of *T. pyogenes*.

We found that the susceptibility of *msrA*-positive *T. pyogenes* varied after luteolin treatment. It is worth noting that the MICs of macrolides did not change after luteolin treatment for strain BM-H06-3. Our previous study showed that there were two site mutations (A753T and G754T) in the 23S rRNA II region of strain BM-H06-3, which also mediated high-level resistance to macrolides. This may be the reason why luteolin failed to reverse the resistance of strain BM-H06-3. However, after reserpine treatment, the MICs of the four macrolides against strain BM-H06-3 decreased, which indicated that the action mechanisms of luteolin and reserpine as efflux pump inhibitors were different.

Efflux pumps can be inhibited by EPIs in different ways: (i) downregulating the expression of the efflux pump gene, (ii) interfering with the assembly of efflux pump proteins, (iii) blocking the efflux pump to avoid substrate binding to the active site, and (iv) blocking the energy supply so that the efflux pump loses its energy source [40]. It has been reported that MRSA treated with silybin for 16 h showed a 36% and 49% reduction in the expression of the *norA* and *qacA*/*B* genes and became sensitive to ciprofloxacin [35]. Ketoconazole could increase the susceptibility of *Staphylococcus aureus* to fluoroquinolones, and the inhibition of the expression of *norA*, which encodes an efflux pump, may contribute to this phenomenon [15]. In this study, the qRT–PCR results showed that luteolin could significantly inhibit the expression of the *msrA* gene (Figure 2). Interestingly, we found that in some strains (HC03-1, HC-H03-3, HC-H02-2, BM07-1, and RY04-2), the inhibitory effect of luteolin on *msrA* gene expression was stronger than that of reserpine, while in some strains (BM-H06-3, BM-H11-1, and BM-H01-1), the opposite trend was observed. This distinction may be due to the differences in their mechanisms of action on the inhibition of *msrA* gene transcription.

We further verified whether luteolin affected the expression of the MsrA protein. As shown in Figure 4, luteolin significantly downregulated the expression of the MsrA protein in *T. pyogenes*. Although the effects of luteolin and reserpine on *msrA* gene expression were irregular, the expression level of the MsrA protein in all *msrA*-positive isolates after luteolin treatment was lower than that after reserpine treatment. These results indicate that luteolin may also act on the posttranscriptional processing of the *msrA* gene, resulting in lower expression of MsrA protein in comparison with that in cells treated with reserpine.

Some efflux pump inhibitors can bind efflux pumps, resulting in a reduced ability of the efflux pumps to interact with their substrates [41]. Crystallization studies have shown that verapamil, a typical EPI, could bind to the active site of the MATE efflux pumps, similar to the substrates of the pumps [42]. Quercetin, a flavonoid compound, could stably bind to the Mmr pump in *Mycobacterium tuberculosis* and the EmrE pump in *Escherichia coli* [43]. In this study, the results of the affinity test revealed that luteolin had a reliable binding ability with MsrA (Figure 5). We further used molecular docking technology to analyse the binding site of luteolin with MsrA. The results showed that luteolin lies in the active pocket of MsrA and interacts with amino acid residues mainly by hydrogen bonds (Figure 6). Importantly, we found that most of the amino acid residues (Gly39, Asn40, Gly41, Thr42, Gly43, and Lys44) interacting with luteolin were located in the ATP-binding and ATPase active regions of MsrA. This binding probably caused MsrA, an ATP-dependent efflux pump, to lose its ability to discharge substrates due to a lack of energy supply. Tariquidar is a third-generation P-glycoprotein inhibitor that binds to the ATP-binding site of P-glycoprotein and inhibits the efflux pump by inhibiting ATPase activity [44]. Luteolin may share a similar mechanism with tariquidar.

In conclusion, this study indicated that luteolin is a potential efflux pump inhibitor that can increase the susceptibility of *T. pyogenes* to macrolides by targeting the MsrA efflux pump. The results of this research showed that luteolin inhibits the activity of the MsrA efflux pump of *T. pyogenes* mainly through the following mechanisms: (i) inhibiting the expression of the *msrA* gene and MsrA protein and (ii) interacting with ATP-binding and ATPase activity regions of the MsrA efflux pump and blocking the energy acquisition of the MsrA efflux pump. Therefore, luteolin may be a potential EPI for use in combating infections caused by antimicrobial-resistant bacteria.

## Data Availability

The datasets used and/or analysed during the current study are included in this article or are available from the corresponding author on reasonable request.
